# Adenosine-perfusion at 1.5 Tesla is superior to 3 Tesla for the detection of coronary artery disease

**DOI:** 10.1186/1532-429X-15-S1-P187

**Published:** 2013-01-30

**Authors:** Thomas Walcher, Katharina Ikuye, Wolfgang Rottbauer, Jochen Wöhrle, Peter Bernhardt

**Affiliations:** 1 Department of Internal Medicine II, Cardiology, Ulm, Germany

## Background

To compare a compiled clinical routine cardiac magnetic resonance imaging (CMR) protocol performed at both 1.5-T and 3.0-T in patients with suspected coronary artery disease (CAD) undergoing coronary x-ray angiography.

CMR including adenosine perfusion and late gadolinium enhancement (LGE) at 1.5-T has been established for noninvasive detection of relevant CAD. However, little is known about the potential advantages of 3.0-T to detect CAD.

## Methods

Fifty-two evaluable patients (62.3 ± 10.2 years) were included into the study. All patients were scanned at both 1.5-T and 3.0-T including adenosine stress and rest perfusion, and LGE imaging. CMR images were analyzed by two blinded readers in consensus. A significant CAD was diagnosed by quantitative coronary analysis.

## Results

Diagnostic accuracy of the combined analysis of perfusion and LGE imaging yielded better values at 1.5-T and 3.0-T than the analysis of perfusion images alone. Specificity and sensitivity at 3.0-T was superior to 1.5-T in detecting coronary stenoses ≥50% (90% vs.75% and 84.4% vs.75%) and ≥70% (88% vs. 80% and 96.3% vs. 88.9%).

**Figure 1:**
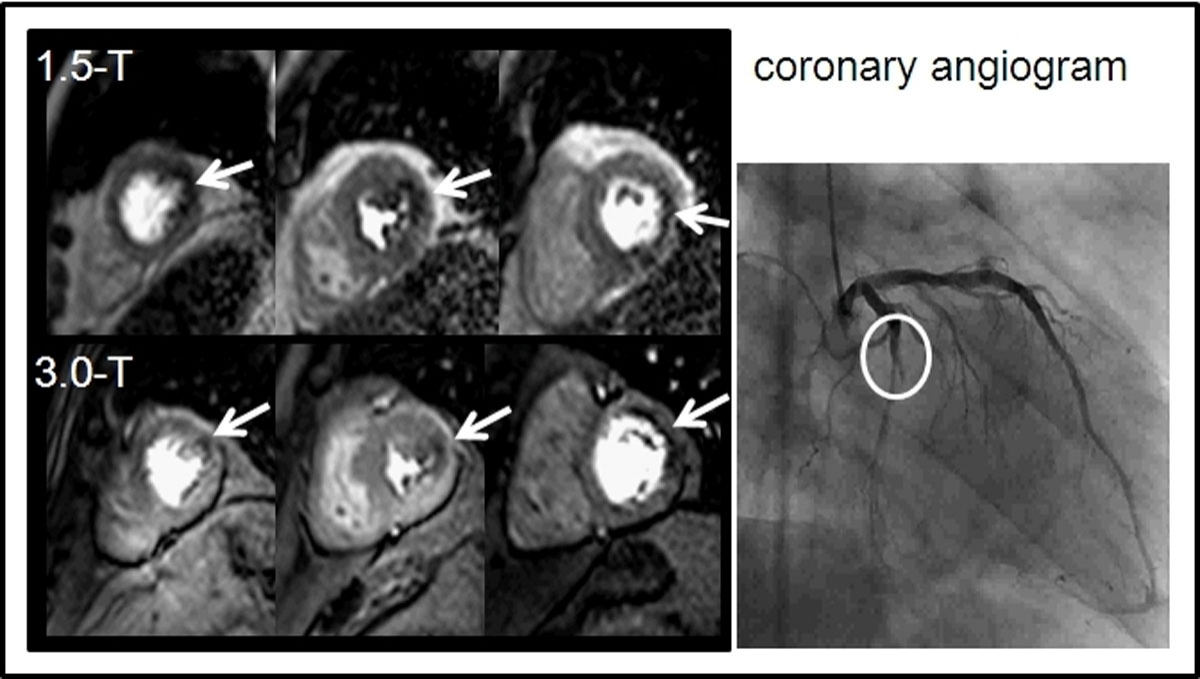
Example of an adenosine perfusion CMR examination at 1.5-T and 3.0-T revealing a lateral wall perfusion deficit (arrows) consistent with an occlusion of the LCX (circle) as seen on coronary angiogram.

**Figure 2:**
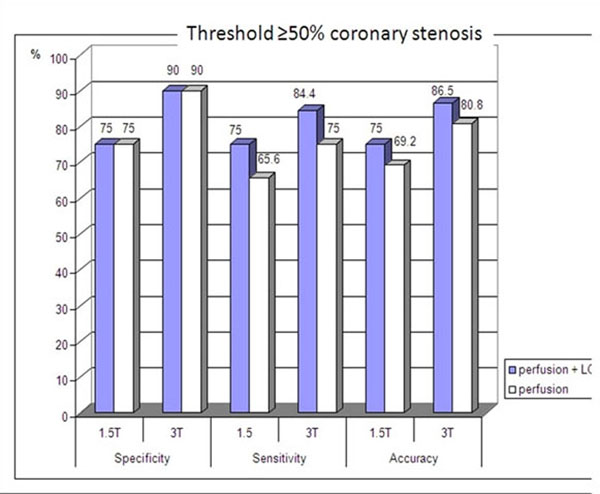
Bar diagram comparing both analysis algorithms (perfusion vs. perfusion + LGE analysis) and both field strengths (1.5 vs. 3 T) for diagnostic accuracy regarding a threshold of ≥50% coronary artery stenosis.

**Figure 3:**
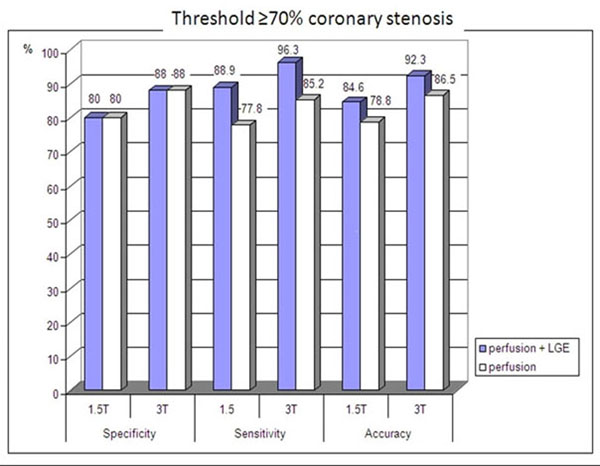
Bar diagram comparing both analysis algorithms (perfusion vs. perfusion + LGE analysis) and both field strengths (1.5 vs. 3 T) for diagnostic accuracy regarding a threshold of ≥70% coronary artery stenosis

## Conclusions

This study showed that CMR at 3.0-T in a routine clinical setting is superior to 1.5-T in detection of significant CAD. 3.0-T might become the preferred CMR field strength for evaluation of CAD in clinical practice.

## Funding

This study was partly funded by a research grant of Guerbet, France.

